# Supplementary material for: Combining growth-promoting genes leads to positive epistasis in Arabidopsis thaliana
Source: eLife. 2014 Apr 29;3:e02252. doi: 10.7554/eLife.02252 (PMC4014012; doi:10.7554/eLife.02252)
Supplement: Supplementary file 1. — Column ‘line’: heterozygous combination, Column ‘% to parA’: if a combination A is combined with B, par A represents the parent A, Column ‘% to parB’: if a combination A is combined with B, parB represents the parent B, Column ‘Pcross-parA’: pvalue, Column ‘Pcross-parB’: pvalue. DOI: http://dx.doi.org/10.7554/eLife.02252.081 [file elife02252s001.pdf]

Line	% to parA	% to parB	Pcross-parA	Pcross-parB
**AN3_ANT**	103	104	1,80E-01	1,70E-01
**AN3_AVP1**	114	100	1,00E-04	8,60E-01
**AN3_BRI1**	101	98	6,00E-01	3,10E-01
**AN3_DA1**	99	98	6,10E-01	6,60E-01
**AN3_EOD**	101	114	5,80E-01	1,00E-04
**AN3_EXP10**	104	112	1,40E-01	4,00E-04
**AN3_GA20OX**	114	112	1,00E-04	1,00E-04
**AN3_GRF5**	101	115	4,90E-02	1,00E-04
**AN3_JAW**	71	98	1,00E-04	3,80E-01
**AN3_PPD**	101	93	7,50E-01	1,50E-02
**AN3_SAUR**	103	103	3,00E-01	2,60E-01
**ANT_AVP1**	119	113	1,00E-04	1,00E-04
**ANT_BRI1**	107	113	1,00E-01	2,00E-04
**ANT_DA1**	95	105	3,00E-01	7,90E-01
**ANT_EOD**	108	129	4,90E-02	1,00E-04
**ANT_EXP10**	101	118	8,00E-01	1,00E-04
**ANT_GRF5**	94	117	4,70E-01	1,00E-04
**ANT_PPD**	98	97	5,90E-01	4,80E-01
**ANT_SAUR**	123	133	1,00E-04	1,00E-04
**AVP1_BRI1**	109	111	9,00E-04	9,00E-04
**AVP1_DA1**	106	109	3,70E-02	6,00E-04
**AVP1_EOD**	101	115	9,20E-01	1,00E-04
**AVP1_EXP10**	106	118	1,90E-02	1,00E-04
**AVP1_PPD**	94	90	1,20E-02	2,00E-04
**BRI1_DA1**	135	117	1,00E-04	1,00E-04
**BRI1_EOD**	138	133	1,00E-04	1,00E-04
**BRI1_EXP10**	128	119	1,00E-04	1,00E-04
**BRI1_GA20OX**	117	100	1,00E-04	9,40E-01
**BRI1_GRF5**	121	120	1,00E-04	1,00E-04
**BRI1_PPD**	130	105	1,00E-04	9,30E-02
**BRI1_SAUR**	137	119	1,00E-04	1,00E-04
**DA1_EXP10**	100	136	9,30E-01	1,00E-04
**DA1_GA20OX**	105	130	9,50E-02	1,00E-04
**DA1_GRF5**	80	115	1,00E-04	1,00E-04
**DA1_JAW**	50	87	1,00E-04	1,00E-04
**DA1_PPD**	90	106	1,00E-04	4,40E-02
**DA1_SAUR**	98	124	3,90E-01	1,00E-04
**EOD_PPD**	88	92	1,00E-04	2,50E-03
**EXP10_GRF5**	75	87	1,00E-04	1,00E-04
**EXP10_JAW**	58	79	1,00E-04	1,00E-04
**EXP10_PPD**	99	92	5,50E-01	7,70E-03
**EXP10_SAUR**	105	106	8,70E-02	2,80E-02
**GA20OX_GRF5**	98	121	4,80E-01	1,00E-04

**GA20OX_JAW**	58	87	1,00E-04	1,00E-04
**GA20OX_PPD**	107	109	4,50E-02	7,00E-03
**GA20OX_SAUR**	106	116	8,60E-01	5,00E-04
**GRF5_JAW**	53	71	1,00E-04	1,00E-04
**GRF5_PPD**	100	91	9,70E-01	7,30E-03
**GRF5_SAUR**	136	135	1,00E-04	1,00E-04
**JAW_PPD**	117	74	1,00E-04	1,00E-04
**JAW_SAUR**	116	79	1,00E-04	1,00E-04
**PPD_SAUR**	129	115	1,00E-04	1,00E-04
**SAMBA_AN3**	110	116	1,00E-03	1,00E-04
**SAMBA_ANT**	115	115	1,00E-04	1,00E-04
**SAMBA_AVP1**	119	102	1,00E-04	4,20E-01
**SAMBA_BRI1**	107	103	1,50E-02	3,40E-01
**SAMBA_DA1**	115	113	1,00E-04	1,00E-04
**SAMBA_EOD**	113	125	1,00E-04	1,00E-04
**SAMBA_EXP10**	108	115	2,90E-02	1,00E-04
**SAMBA_PPD**	99	91	8,10E-01	7,80E-03
**SAMBA_SAUR**	117	116	1,00E-04	1,00E-04
