# Supplementary material for: Combining growth-promoting genes leads to positive epistasis in Arabidopsis thaliana
Source: eLife. 2014 Apr 29;3:e02252. doi: 10.7554/eLife.02252 (PMC4014012; doi:10.7554/eLife.02252)
Supplement: Supplementary file 2. — DOI: http://dx.doi.org/10.7554/eLife.02252.082 [file elife02252s002.pdf]

	Forward	Reverse
AN3	CAGGGAGAAGGAGGGTCACAC	ATTGAAGATCGAGCCGCCATTAG
ANT	AGGTGGTTCCAACAAAGAAGTCAG	GACGGTCTTAAGCTCAGCATTAGG
AVP1	GCAGGCAGTTCAACACCATCC	GCATGACAAGGCAACCAGGAG
BRI1	AAAGTTGCGGTTGCGTGTTTG	GTTGACTGTGAATCTATCCCTGACC
EOD	GGGCTCCTCCTCTTCTTTTC	CACACAAGCGAAAATGGAAC
EXP10	CTTCAACGCCGCTCCTGCC	GATCGCCGCCGAGTGAACG
GA20OX1	CATCAACGTTCTCGAGCTTGATGTTC	GCGGCTCGTGTATTCATGAGCG
GRF5	TCAGTTCAATGTCTTAGCCTCTGC	CCCAACTCCTCCAACTCTCTCC
JAW	TCCAATTTTCTTGATTAATCTTTCCTGC	CACCTATCCATGGCGATGCCTTA
PPD2	CCGGTCTATCATGCATTTTG	AAGCAGGTGCCTTTTCAAGT
SAMBA	ACGAAGCTATGCGTGTTTTG	GGACCTTCGAACATCCAACT
SAUR19	GAAGATTCTAAGCCGCTCCA	TGACGGTTGGCTCAAGTATG
