# Supplementary material for: Combining growth-promoting genes leads to positive epistasis in Arabidopsis thaliana
Source: eLife. 2014 Apr 29;3:e02252. doi: 10.7554/eLife.02252 (PMC4014012; doi:10.7554/eLife.02252)
Supplement: Supplementary file 3. — DOI: http://dx.doi.org/10.7554/eLife.02252.083 [file elife02252s003.pdf]

**Cross**	Gene combination
**Genotype**	Genotype within the combination, ordered as follows: cross, parent 1, parent 2, reference
	Proportion of the Variance attributed by the different factors in the model. To calcute this, all factors were treated as random factors. These values are determined per gene combination and are therefore the same for each of the Genotypes per combination.
**VarE**	Proportion of the variance explained by the factor Experiment
**VarG**	Proportion of the variance explained by the factor Genotype
**VarGxE**	Proportion of the variance explained by the interaction of Genotype and Experiment
**VarResidual**	Residual Variance
**Obs/Exp (selected)**	The rosette area was estimated by Least Square Means (LSM) based on the applied mixed model. We included the interaction of Genotype and Experiment (GxE) in the model when appropriate. Here we provide the ratio of Observed and Expected (calculated using a multiplicative model) of both the model with and without interaction.
**Obs/Exp (interaction)**	LSM calculated using the mixed model with interaction of GxE included
**Obs/Exp (without interaction)**	LSM calculated using the mixed model without interaction terms
**P interaction**	p-value for the significance of the GxE interaction, based on a likelihood ratio test
**P synergistic**	FDR corrected p-value for synergistic effect (see Material and Methods for model details)

Cross	Genotype	VarE	VarG	VarGxE	VarResidual	Obs/Exp (selected)	Obs/Exp (interaction)	Obs/Exp (without interaction)	P interaction	P synergistic
BRI1_EOD	BRI1_EOD	0	59.55	1.39	39.05	47.74	47.84	47.74	2.10E-01	0.0001
BRI1_EOD	BRI1_COL	0	59.55	1.39	39.05	47.74	47.84	47.74	2.10E-01	0.0001
BRI1_EOD	COL_EOD	0	59.55	1.39	39.05	47.74	47.84	47.74	2.10E-01	0.0001
BRI1_EOD	COL	0	59.55	1.39	39.05	47.74	47.84	47.74	2.10E-01	0.0001
BRI1_EXP10	BRI1_EXP10	0.15	36.35	11.4	52.11	33.03	33.03	35.46	9.20E-03	0.0001
BRI1_EXP10	BRI1_COL	0.15	36.35	11.4	52.11	33.03	33.03	35.46	9.20E-03	0.0001
BRI1_EXP10	COL_EXP10	0.15	36.35	11.4	52.11	33.03	33.03	35.46	9.20E-03	0.0001
BRI1_EXP10	COL	0.15	36.35	11.4	52.11	33.03	33.03	35.46	9.20E-03	0.0001
BRI1_GRF5	BRI1_GRF5	0.75	35.15	3.12	60.98	32.93	32.63	32.93	1.00E-01	0.0001
BRI1_GRF5	BRI1_COL	0.75	35.15	3.12	60.98	32.93	32.63	32.93	1.00E-01	0.0001
BRI1_GRF5	COL_GRF5	0.75	35.15	3.12	60.98	32.93	32.63	32.93	1.00E-01	0.0001
BRI1_GRF5	COL	0.75	35.15	3.12	60.98	32.93	32.63	32.93	1.00E-01	0.0001
BRI1_SAUR	BRI1_SAUR	0.79	51.56	1.34	46.31	31.89	32.30	31.89	5.80E-01	0.0001
BRI1_SAUR	BRI1_COL	0.79	51.56	1.34	46.31	31.89	32.30	31.89	5.80E-01	0.0001
BRI1_SAUR	COL_SAUR	0.79	51.56	1.34	46.31	31.89	32.30	31.89	5.80E-01	0.0001
BRI1_SAUR	COL	0.79	51.56	1.34	46.31	31.89	32.30	31.89	5.80E-01	0.0001
GRF5_SAUR	GRF5_SAUR	2.55	57.7	3.89	35.86	31.44	30.75	31.44	5.80E-02	0.0001
GRF5_SAUR	GRF5_COL	2.55	57.7	3.89	35.86	31.44	30.75	31.44	5.80E-02	0.0001
GRF5_SAUR	COL_SAUR	2.55	57.7	3.89	35.86	31.44	30.75	31.44	5.80E-02	0.0001
GRF5_SAUR	COL	2.55	57.7	3.89	35.86	31.44	30.75	31.44	5.80E-02	0.0001
BRI1_DA1	BRI1_DA1	0	50.48	4.37	45.14	30.09	30.09	31.24	4.20E-02	0.0001
BRI1_DA1	BRI1_COL	0	50.48	4.37	45.14	30.09	30.09	31.24	4.20E-02	0.0001
BRI1_DA1	COL_DA1	0	50.48	4.37	45.14	30.09	30.09	31.24	4.20E-02	0.0001
BRI1_DA1	COL	0	50.48	4.37	45.14	30.09	30.09	31.24	4.20E-02	0.0001
PPD_SAUR	PPD_SAUR	2.78	42.78	3.44	51	24.14	24.51	24.14	1.80E-01	0.0001
PPD_SAUR	PPD_COL	2.78	42.78	3.44	51	24.14	24.51	24.14	1.80E-01	0.0001
PPD_SAUR	COL_SAUR	2.78	42.78	3.44	51	24.14	24.51	24.14	1.80E-01	0.0001
PPD_SAUR	COL	2.78	42.78	3.44	51	24.14	24.51	24.14	1.80E-01	0.0001

SAMBA_EOD	SAMBA_EOD	0	35.23	4.02	60.75	20.92	19.14	20.92	4.30E-01	0.0001
SAMBA_EOD	SAMBA_COL	0	35.23	4.02	60.75	20.92	19.14	20.92	4.30E-01	0.0001
SAMBA_EOD	COL_EOD	0	35.23	4.02	60.75	20.92	19.14	20.92	4.30E-01	0.0001
SAMBA_EOD	COL	0	35.23	4.02	60.75	20.92	19.14	20.92	4.30E-01	0.0001
ANT_SAUR	ANT_SAUR	1.13	59.43	1.87	37.56	18.32	17.86	18.32	6.50E-01	0.0007
ANT_SAUR	ANT_COL	1.13	59.43	1.87	37.56	18.32	17.86	18.32	6.50E-01	0.0007
ANT_SAUR	COL_SAUR	1.13	59.43	1.87	37.56	18.32	17.86	18.32	6.50E-01	0.0007
ANT_SAUR	COL	1.13	59.43	1.87	37.56	18.32	17.86	18.32	6.50E-01	0.0007
BRI1_PPD	BRI1_PPD	0	49.25	1.09	49.66	16.13	16.59	16.13	3.30E-01	0.0001
BRI1_PPD	BRI1_COL	0	49.25	1.09	49.66	16.13	16.59	16.13	3.30E-01	0.0001
BRI1_PPD	COL_PPD	0	49.25	1.09	49.66	16.13	16.59	16.13	3.30E-01	0.0001
BRI1_PPD	COL	0	49.25	1.09	49.66	16.13	16.59	16.13	3.30E-01	0.0001
ANT_EOD	ANT_EOD	1.88	48.86	0	49.26	15.01	15.01	15.01	0.00E+00	0.0017
ANT_EOD	ANT_COL	1.88	48.86	0	49.26	15.01	15.01	15.01	0.00E+00	0.0017
ANT_EOD	COL_EOD	1.88	48.86	0	49.26	15.01	15.01	15.01	0.00E+00	0.0017
ANT_EOD	COL	1.88	48.86	0	49.26	15.01	15.01	15.01	0.00E+00	0.0017
SAMBA_SAUR	SAMBA_SAUR	0	34.64	4.45	60.91	12.55	12.86	12.55	1.30E-01	0.0002
SAMBA_SAUR	SAMBA_COL	0	34.64	4.45	60.91	12.55	12.86	12.55	1.30E-01	0.0002
SAMBA_SAUR	COL_SAUR	0	34.64	4.45	60.91	12.55	12.86	12.55	1.30E-01	0.0002
SAMBA_SAUR	COL	0	34.64	4.45	60.91	12.55	12.86	12.55	1.30E-01	0.0002
SAMBA_AN3	SAMBA_AN3	1.48	10.74	15.84	71.94	12.53	9.42	12.53	5.70E-02	0.0014
SAMBA_AN3	SAMBA_COL	1.48	10.74	15.84	71.94	12.53	9.42	12.53	5.70E-02	0.0014
SAMBA_AN3	COL_AN3	1.48	10.74	15.84	71.94	12.53	9.42	12.53	5.70E-02	0.0014
SAMBA_AN3	COL	1.48	10.74	15.84	71.94	12.53	9.42	12.53	5.70E-02	0.0014
JAW_SAUR	JAW_SAUR	0.98	59.22	3.32	36.48	12.05	12.77	12.05	9.20E-02	0.0067
JAW_SAUR	JAW_COL	0.98	59.22	3.32	36.48	12.05	12.77	12.05	9.20E-02	0.0067
JAW_SAUR	COL_SAUR	0.98	59.22	3.32	36.48	12.05	12.77	12.05	9.20E-02	0.0067
JAW_SAUR	COL	0.98	59.22	3.32	36.48	12.05	12.77	12.05	9.20E-02	0.0067
SAMBA_EXP10	SAMBA_EXP10	0	12.83	2.3	84.87	11.18	10.81	11.18	5.20E-01	0.018
SAMBA_EXP10	SAMBA_COL	0	12.83	2.3	84.87	11.18	10.81	11.18	5.20E-01	0.018
SAMBA_EXP10	COL_EXP10	0	12.83	2.3	84.87	11.18	10.81	11.18	5.20E-01	0.018

SAMBA_EXP10	COL	0	12.83	2.3	84.87	11.18	10.81	11.18	5.20E-01	0.018
SAMBA_ANT	SAMBA_ANT	0	27.22	2.62	70.16	10.96	10.08	10.96	2.90E-01	0.0053
SAMBA_ANT	SAMBA_COL	0	27.22	2.62	70.16	10.96	10.08	10.96	2.90E-01	0.0053
SAMBA_ANT	COL_ANT	0	27.22	2.62	70.16	10.96	10.08	10.96	2.90E-01	0.0053
SAMBA_ANT	COL	0	27.22	2.62	70.16	10.96	10.08	10.96	2.90E-01	0.0053
AN3_GRF5	AN3_GRF5	6.22	5.67	29.14	58.97	10.6	10.60	16.62	1.10E-04	0.0001
AN3_GRF5	AN3_COL	6.22	5.67	29.14	58.97	10.6	10.60	16.62	1.10E-04	0.0001
AN3_GRF5	COL_GRF5	6.22	5.67	29.14	58.97	10.6	10.60	16.62	1.10E-04	0.0001
AN3_GRF5	COL	6.22	5.67	29.14	58.97	10.6	10.60	16.62	1.10E-04	0.0001
SAMBA_DA1	SAMBA_DA1	3.7	26.56	0	69.74	10.56	10.56	10.56	1.00E+00	0.0083
SAMBA_DA1	SAMBA_COL	3.7	26.56	0	69.74	10.56	10.56	10.56	1.00E+00	0.0083
SAMBA_DA1	COL_DA1	3.7	26.56	0	69.74	10.56	10.56	10.56	1.00E+00	0.0083
SAMBA_DA1	COL	3.7	26.56	0	69.74	10.56	10.56	10.56	1.00E+00	0.0083
BRI1_GA20OX	BRI1_GA20OX	2.06	24.08	0.24	73.62	10.39	10.45	10.39	9.30E-01	0.0052
BRI1_GA20OX	BRI1_COL	2.06	24.08	0.24	73.62	10.39	10.45	10.39	9.30E-01	0.0052
BRI1_GA20OX	COL_GA20OX	2.06	24.08	0.24	73.62	10.39	10.45	10.39	9.30E-01	0.0052
BRI1_GA20OX	COL	2.06	24.08	0.24	73.62	10.39	10.45	10.39	9.30E-01	0.0052
AVP1_EXP10	AVP1_EXP10	0	24.34	13.97	61.69	10.27	10.27	9.19	1.50E-04	0.0063
AVP1_EXP10	AVP1_COL	0	24.34	13.97	61.69	10.27	10.27	9.19	1.50E-04	0.0063
AVP1_EXP10	COL_EXP10	0	24.34	13.97	61.69	10.27	10.27	9.19	1.50E-04	0.0063
AVP1_EXP10	COL	0	24.34	13.97	61.69	10.27	10.27	9.19	1.50E-04	0.0063
AN3_EOD	AN3_EOD	5.05	18.2	0	76.75	8.74	8.74	8.74	0.00E+00	0.016
AN3_EOD	AN3_COL	5.05	18.2	0	76.75	8.74	8.74	8.74	0.00E+00	0.016
AN3_EOD	COL_EOD	5.05	18.2	0	76.75	8.74	8.74	8.74	0.00E+00	0.016
AN3_EOD	COL	5.05	18.2	0	76.75	8.74	8.74	8.74	0.00E+00	0.016
AVP1_EOD	AVP1_EOD	0	18.79	13.64	67.58	7.93	7.93	7.14	1.80E-03	0.036
AVP1_EOD	AVP1_COL	0	18.79	13.64	67.58	7.93	7.93	7.14	1.80E-03	0.036
AVP1_EOD	COL_EOD	0	18.79	13.64	67.58	7.93	7.93	7.14	1.80E-03	0.036
AVP1_EOD	COL	0	18.79	13.64	67.58	7.93	7.93	7.14	1.80E-03	0.036
AN3_EXP10	AN3_EXP10	1.68	11.49	12.93	73.9	7.77	10.33	7.77	7.10E-02	0.066
AN3_EXP10	AN3_COL	1.68	11.49	12.93	73.9	7.77	10.33	7.77	7.10E-02	0.066

AN3_EXP10	COL_EXP10	1.68	11.49	12.93	73.9	7.77	10.33	7.77	7.10E-02	0.066
AN3_EXP10	COL	1.68	11.49	12.93	73.9	7.77	10.33	7.77	7.10E-02	0.066
AN3_GA20OX	AN3_GA20OX	0	28.04	1.35	70.6	7.27	7.37	7.27	4.40E-01	0.035
AN3_GA20OX	AN3_COL	0	28.04	1.35	70.6	7.27	7.37	7.27	4.40E-01	0.035
AN3_GA20OX	COL_GA20OX	0	28.04	1.35	70.6	7.27	7.37	7.27	4.40E-01	0.035
AN3_GA20OX	COL	0	28.04	1.35	70.6	7.27	7.37	7.27	4.40E-01	0.035
GA20OX_GRF5	GA20OX_GRF5	0.9	35.6	7.59	55.91	6.97	6.97	6.9	7.70E-03	0.075
GA20OX_GRF5	GA20OX_COL	0.9	35.6	7.59	55.91	6.97	6.97	6.9	7.70E-03	0.075
GA20OX_GRF5	COL_GRF5	0.9	35.6	7.59	55.91	6.97	6.97	6.9	7.70E-03	0.075
GA20OX_GRF5	COL	0.9	35.6	7.59	55.91	6.97	6.97	6.9	7.70E-03	0.075
JAW_PPD	JAW_PPD	0.32	66.74	2.16	30.78	5.35	5.35	6.4	4.30E-02	0.11
JAW_PPD	JAW_COL	0.32	66.74	2.16	30.78	5.35	5.35	6.4	4.30E-02	0.11
JAW_PPD	COL_PPD	0.32	66.74	2.16	30.78	5.35	5.35	6.4	4.30E-02	0.11
JAW_PPD	COL	0.32	66.74	2.16	30.78	5.35	5.35	6.4	4.30E-02	0.11
ANT_EXP10	ANT_EXP10	2.21	34.5	0	63.29	3.96	3.96	3.96	1.00E+00	0.38
ANT_EXP10	ANT_COL	2.21	34.5	0	63.29	3.96	3.96	3.96	1.00E+00	0.38
ANT_EXP10	COL_EXP10	2.21	34.5	0	63.29	3.96	3.96	3.96	1.00E+00	0.38
ANT_EXP10	COL	2.21	34.5	0	63.29	3.96	3.96	3.96	1.00E+00	0.38
AVP1_BRI1	AVP1_BRI1	6.66	19.52	12.1	61.72	3.06	3.06	0.81	1.20E-05	0.8
AVP1_BRI1	AVP1_COL	6.66	19.52	12.1	61.72	3.06	3.06	0.81	1.20E-05	0.8
AVP1_BRI1	COL_BRI1	6.66	19.52	12.1	61.72	3.06	3.06	0.81	1.20E-05	0.8
AVP1_BRI1	COL	6.66	19.52	12.1	61.72	3.06	3.06	0.81	1.20E-05	0.8
DA1_EXP10	DA1_EXP10	0.84	68.22	1.67	29.27	3.04	2.83	3.04	3.80E-01	0.34
DA1_EXP10	DA1_COL	0.84	68.22	1.67	29.27	3.04	2.83	3.04	3.80E-01	0.34
DA1_EXP10	COL_EXP10	0.84	68.22	1.67	29.27	3.04	2.83	3.04	3.80E-01	0.34
DA1_EXP10	COL	0.84	68.22	1.67	29.27	3.04	2.83	3.04	3.80E-01	0.34
GA20OX_SAUR	GA20OX_SAUR	0	20.06	25.37	54.57	2.46	2.46	-4.35	3.10E-06	0.23
GA20OX_SAUR	GA20OX_COL	0	20.06	25.37	54.57	2.46	2.46	-4.35	3.10E-06	0.23
GA20OX_SAUR	COL_SAUR	0	20.06	25.37	54.57	2.46	2.46	-4.35	3.10E-06	0.23
GA20OX_SAUR	COL	0	20.06	25.37	54.57	2.46	2.46	-4.35	3.10E-06	0.23
ANT_GRF5	ANT_GRF5	0	34.53	9.47	56.01	2.38	2.38	5.88	1.70E-02	0.23

ANT_GRF5	ANT_COL	0	34.53	9.47	56.01	2.38	2.38	5.88	1.70E-02	0.23
ANT_GRF5	COL_GRF5	0	34.53	9.47	56.01	2.38	2.38	5.88	1.70E-02	0.23
ANT_GRF5	COL	0	34.53	9.47	56.01	2.38	2.38	5.88	1.70E-02	0.23
AVP1_DA1	AVP1_DA1	0	13.05	17.28	69.66	1.9	1.90	2.04	5.70E-05	0.53
AVP1_DA1	AVP1_COL	0	13.05	17.28	69.66	1.9	1.90	2.04	5.70E-05	0.53
AVP1_DA1	COL_DA1	0	13.05	17.28	69.66	1.9	1.90	2.04	5.70E-05	0.53
AVP1_DA1	COL	0	13.05	17.28	69.66	1.9	1.90	2.04	5.70E-05	0.53
EXP10_SAUR	EXP10_SAUR	0.25	8.74	5.16	85.86	0.74	1.35	0.74	4.20E-01	0.81
EXP10_SAUR	EXP10_COL	0.25	8.74	5.16	85.86	0.74	1.35	0.74	4.20E-01	0.81
EXP10_SAUR	COL_SAUR	0.25	8.74	5.16	85.86	0.74	1.35	0.74	4.20E-01	0.81
EXP10_SAUR	COL	0.25	8.74	5.16	85.86	0.74	1.35	0.74	4.20E-01	0.81
SAMBA_AVP1	SAMBA_AVP1	3.72	41.26	2.3	52.73	-0.29	-0.95	-0.29	5.80E-01	0.92
SAMBA_AVP1	SAMBA_COL	3.72	41.26	2.3	52.73	-0.29	-0.95	-0.29	5.80E-01	0.92
SAMBA_AVP1	COL_AVP1	3.72	41.26	2.3	52.73	-0.29	-0.95	-0.29	5.80E-01	0.92
SAMBA_AVP1	COL	3.72	41.26	2.3	52.73	-0.29	-0.95	-0.29	5.80E-01	0.92
AN3_ANT	AN3_ANT	3.88	6.9	2.67	86.55	-0.9	-0.35	-0.9	4.50E-01	0.76
AN3_ANT	AN3_COL	3.88	6.9	2.67	86.55	-0.9	-0.35	-0.9	4.50E-01	0.76
AN3_ANT	COL_ANT	3.88	6.9	2.67	86.55	-0.9	-0.35	-0.9	4.50E-01	0.76
AN3_ANT	COL	3.88	6.9	2.67	86.55	-0.9	-0.35	-0.9	4.50E-01	0.76
AN3_SAUR	AN3_SAUR	3.5	3.58	5.24	87.68	-1.14	-0.77	-1.14	2.60E-01	0.7
AN3_SAUR	AN3_COL	3.5	3.58	5.24	87.68	-1.14	-0.77	-1.14	2.60E-01	0.7
AN3_SAUR	COL_SAUR	3.5	3.58	5.24	87.68	-1.14	-0.77	-1.14	2.60E-01	0.7
AN3_SAUR	COL	3.5	3.58	5.24	87.68	-1.14	-0.77	-1.14	2.60E-01	0.7
SAMBA_BRI1	SAMBA_BRI1	0	13.16	2.34	84.5	-1.14	-0.44	-1.14	2.10E-01	0.73
SAMBA_BRI1	SAMBA_COL	0	13.16	2.34	84.5	-1.14	-0.44	-1.14	2.10E-01	0.73
SAMBA_BRI1	COL_BRI1	0	13.16	2.34	84.5	-1.14	-0.44	-1.14	2.10E-01	0.73
SAMBA_BRI1	COL	0	13.16	2.34	84.5	-1.14	-0.44	-1.14	2.10E-01	0.73
ANT_BRI1	ANT_BRI1	0	31.61	3.07	65.32	-1.31	-0.86	-1.31	1.20E-01	0.77
ANT_BRI1	ANT_COL	0	31.61	3.07	65.32	-1.31	-0.86	-1.31	1.20E-01	0.77
ANT_BRI1	COL_BRI1	0	31.61	3.07	65.32	-1.31	-0.86	-1.31	1.20E-01	0.77
ANT_BRI1	COL	0	31.61	3.07	65.32	-1.31	-0.86	-1.31	1.20E-01	0.77

DA1_GA20OX	DA1_GA20OX	0	63.65	2.51	33.84	-1.36	-1.56	-1.36	1.40E-01	0.68
DA1_GA20OX	DA1_COL	0	63.65	2.51	33.84	-1.36	-1.56	-1.36	1.40E-01	0.68
DA1_GA20OX	COL_GA20OX	0	63.65	2.51	33.84	-1.36	-1.56	-1.36	1.40E-01	0.68
DA1_GA20OX	COL	0	63.65	2.51	33.84	-1.36	-1.56	-1.36	1.40E-01	0.68
ANT_AVP1	ANT_AVP1	0	49.42	12	38.58	-1.6	-1.60	3.08	9.10E-04	0.46
ANT_AVP1	ANT_COL	0	49.42	12	38.58	-1.6	-1.60	3.08	9.10E-04	0.46
ANT_AVP1	COL_AVP1	0	49.42	12	38.58	-1.6	-1.60	3.08	9.10E-04	0.46
ANT_AVP1	COL	0	49.42	12	38.58	-1.6	-1.60	3.08	9.10E-04	0.46
GA20OX_PPD	GA20OX_PPD	0	29.9	3.3	66.8	-4.8	-4.02	-4.8	1.50E-01	0.21
GA20OX_PPD	GA20OX_COL	0	29.9	3.3	66.8	-4.8	-4.02	-4.8	1.50E-01	0.21
GA20OX_PPD	COL_PPD	0	29.9	3.3	66.8	-4.8	-4.02	-4.8	1.50E-01	0.21
GA20OX_PPD	COL	0	29.9	3.3	66.8	-4.8	-4.02	-4.8	1.50E-01	0.21
AN3_AVP1	AN3_AVP1	7.51	35.52	4.15	52.82	-4.94	-5.84	-4.94	1.80E-01	0.11
AN3_AVP1	AN3_COL	7.51	35.52	4.15	52.82	-4.94	-5.84	-4.94	1.80E-01	0.11
AN3_AVP1	COL_AVP1	7.51	35.52	4.15	52.82	-4.94	-5.84	-4.94	1.80E-01	0.11
AN3_AVP1	COL	7.51	35.52	4.15	52.82	-4.94	-5.84	-4.94	1.80E-01	0.11
AN3_DA1	AN3_DA1	0.15	0	15	84.85	-5.27	-5.27	-4.89	1.00E-03	0.17
AN3_DA1	AN3_COL	0.15	0	15	84.85	-5.27	-5.27	-4.89	1.00E-03	0.17
AN3_DA1	COL_DA1	0.15	0	15	84.85	-5.27	-5.27	-4.89	1.00E-03	0.17
AN3_DA1	COL	0.15	0	15	84.85	-5.27	-5.27	-4.89	1.00E-03	0.17
AN3_BRI1	AN3_BRI1	0	2.53	9.56	87.92	-5.49	-5.49	-6.61	1.40E-02	0.055
AN3_BRI1	AN3_COL	0	2.53	9.56	87.92	-5.49	-5.49	-6.61	1.40E-02	0.055
AN3_BRI1	COL_BRI1	0	2.53	9.56	87.92	-5.49	-5.49	-6.61	1.40E-02	0.055
AN3_BRI1	COL	0	2.53	9.56	87.92	-5.49	-5.49	-6.61	1.40E-02	0.055
DA1_SAUR	DA1_SAUR	2.19	56.62	1.55	39.63	-5.95	-6.13	-5.95	4.80E-01	0.066
DA1_SAUR	DA1_COL	2.19	56.62	1.55	39.63	-5.95	-6.13	-5.95	4.80E-01	0.066
DA1_SAUR	COL_SAUR	2.19	56.62	1.55	39.63	-5.95	-6.13	-5.95	4.80E-01	0.066
DA1_SAUR	COL	2.19	56.62	1.55	39.63	-5.95	-6.13	-5.95	4.80E-01	0.066
AN3_JAW	AN3_JAW	0	64.59	5.97	29.44	-5.96	-5.96	-6.34	7.90E-04	0.071
AN3_JAW	AN3_COL	0	64.59	5.97	29.44	-5.96	-5.96	-6.34	7.90E-04	0.071
AN3_JAW	COL_JAW	0	64.59	5.97	29.44	-5.96	-5.96	-6.34	7.90E-04	0.071

AN3_JAW	COL	0	64.59	5.97	29.44	-5.96	-5.96	-6.34	7.90E-04	0.071
ANT_DA1	ANT_DA1	0	17.63	0	82.37	-10.21	-10.21	-10.21	1.00E+00	0.094
ANT_DA1	ANT_COL	0	17.63	0	82.37	-10.21	-10.21	-10.21	1.00E+00	0.094
ANT_DA1	COL_DA1	0	17.63	0	82.37	-10.21	-10.21	-10.21	1.00E+00	0.094
ANT_DA1	COL	0	17.63	0	82.37	-10.21	-10.21	-10.21	1.00E+00	0.094
AN3_PPD	AN3_PPD	2.38	14.23	0	83.39	-10.29	-10.29	-10.29	1.00E+00	0.002
AN3_PPD	AN3_COL	2.38	14.23	0	83.39	-10.29	-10.29	-10.29	1.00E+00	0.002
AN3_PPD	COL_PPD	2.38	14.23	0	83.39	-10.29	-10.29	-10.29	1.00E+00	0.002
AN3_PPD	COL	2.38	14.23	0	83.39	-10.29	-10.29	-10.29	1.00E+00	0.002
GRF5_PPD	GRF5_PPD	0	10.41	2.8	86.79	-10.94	-10.92	-10.94	3.50E-01	0.0053
GRF5_PPD	GRF5_COL	0	10.41	2.8	86.79	-10.94	-10.92	-10.94	3.50E-01	0.0053
GRF5_PPD	COL_PPD	0	10.41	2.8	86.79	-10.94	-10.92	-10.94	3.50E-01	0.0053
GRF5_PPD	COL	0	10.41	2.8	86.79	-10.94	-10.92	-10.94	3.50E-01	0.0053
SAMBA_PPD	SAMBA_PPD	0	6.73	12.66	80.62	-12.03	-9.96	-12.03	8.70E-02	0.0019
SAMBA_PPD	SAMBA_COL	0	6.73	12.66	80.62	-12.03	-9.96	-12.03	8.70E-02	0.0019
SAMBA_PPD	COL_PPD	0	6.73	12.66	80.62	-12.03	-9.96	-12.03	8.70E-02	0.0019
SAMBA_PPD	COL	0	6.73	12.66	80.62	-12.03	-9.96	-12.03	8.70E-02	0.0019
EXP10_PPD	EXP10_PPD	0.22	11.24	2.22	86.32	-12.13	-11.74	-12.13	5.10E-01	0.0003
EXP10_PPD	EXP10_COL	0.22	11.24	2.22	86.32	-12.13	-11.74	-12.13	5.10E-01	0.0003
EXP10_PPD	COL_PPD	0.22	11.24	2.22	86.32	-12.13	-11.74	-12.13	5.10E-01	0.0003
EXP10_PPD	COL	0.22	11.24	2.22	86.32	-12.13	-11.74	-12.13	5.10E-01	0.0003
DA1_GRF5	DA1_GRF5	2	56.63	10.05	31.32	-12.66	-12.66	-11.66	3.10E-07	0.0002
DA1_GRF5	DA1_COL	2	56.63	10.05	31.32	-12.66	-12.66	-11.66	3.10E-07	0.0002
DA1_GRF5	COL_GRF5	2	56.63	10.05	31.32	-12.66	-12.66	-11.66	3.10E-07	0.0002
DA1_GRF5	COL	2	56.63	10.05	31.32	-12.66	-12.66	-11.66	3.10E-07	0.0002
ANT_PPD	ANT_PPD	4.01	15.05	5.62	75.32	-12.94	-13.32	-12.94	3.40E-01	0.0025
ANT_PPD	ANT_COL	4.01	15.05	5.62	75.32	-12.94	-13.32	-12.94	3.40E-01	0.0025
ANT_PPD	COL_PPD	4.01	15.05	5.62	75.32	-12.94	-13.32	-12.94	3.40E-01	0.0025
ANT_PPD	COL	4.01	15.05	5.62	75.32	-12.94	-13.32	-12.94	3.40E-01	0.0025
AVP1_PPD	AVP1_PPD	0.12	11.6	9.49	78.79	-15.63	-15.63	-16.09	6.90E-03	0.0001
AVP1_PPD	AVP1_COL	0.12	11.6	9.49	78.79	-15.63	-15.63	-16.09	6.90E-03	0.0001

AVP1_PPD	COL_PPD	0.12	11.6	9.49	78.79	-15.63	-15.63	-16.09	6.90E-03	0.0001
AVP1_PPD	COL	0.12	11.6	9.49	78.79	-15.63	-15.63	-16.09	6.90E-03	0.0001
EXP10_GRF5	EXP10_GRF5	0	53.64	1.52	44.84	-17.42	-17.61	-17.42	3.80E-01	0.0001
EXP10_GRF5	EXP10_COL	0	53.64	1.52	44.84	-17.42	-17.61	-17.42	3.80E-01	0.0001
EXP10_GRF5	COL_GRF5	0	53.64	1.52	44.84	-17.42	-17.61	-17.42	3.80E-01	0.0001
EXP10_GRF5	COL	0	53.64	1.52	44.84	-17.42	-17.61	-17.42	3.80E-01	0.0001
DA1_PPD	DA1_PPD	0.09	46.84	4.37	48.7	-19.56	-19.06	-19.56	1.80E-01	0.0001
DA1_PPD	DA1_COL	0.09	46.84	4.37	48.7	-19.56	-19.06	-19.56	1.80E-01	0.0001
DA1_PPD	COL_PPD	0.09	46.84	4.37	48.7	-19.56	-19.06	-19.56	1.80E-01	0.0001
DA1_PPD	COL	0.09	46.84	4.37	48.7	-19.56	-19.06	-19.56	1.80E-01	0.0001
EOD_PPD	EOD_PPD	3.83	27.69	0	68.47	-22.03	-22.03	-22.03	1.00E+00	0.0001
EOD_PPD	EOD_COL	3.83	27.69	0	68.47	-22.03	-22.03	-22.03	1.00E+00	0.0001
EOD_PPD	COL_PPD	3.83	27.69	0	68.47	-22.03	-22.03	-22.03	1.00E+00	0.0001
EOD_PPD	COL	3.83	27.69	0	68.47	-22.03	-22.03	-22.03	1.00E+00	0.0001
GA20OX_JAW	GA20OX_JAW	0.12	76.24	6.93	16.7	-24.06	-24.06	-25.73	2.30E-05	0.0001
GA20OX_JAW	GA20OX_COL	0.12	76.24	6.93	16.7	-24.06	-24.06	-25.73	2.30E-05	0.0001
GA20OX_JAW	COL_JAW	0.12	76.24	6.93	16.7	-24.06	-24.06	-25.73	2.30E-05	0.0001
GA20OX_JAW	COL	0.12	76.24	6.93	16.7	-24.06	-24.06	-25.73	2.30E-05	0.0001
EXP10_JAW	EXP10_JAW	0	79.04	2.03	18.93	-24.79	-24.59	-24.79	1.70E-01	0.0001
EXP10_JAW	EXP10_COL	0	79.04	2.03	18.93	-24.79	-24.59	-24.79	1.70E-01	0.0001
EXP10_JAW	COL_JAW	0	79.04	2.03	18.93	-24.79	-24.59	-24.79	1.70E-01	0.0001
EXP10_JAW	COL	0	79.04	2.03	18.93	-24.79	-24.59	-24.79	1.70E-01	0.0001
GRF5_JAW	GRF5_JAW	0.82	84.11	0.23	14.85	-30.41	-30.48	-30.41	8.50E-01	0.0001
GRF5_JAW	GRF5_COL	0.82	84.11	0.23	14.85	-30.41	-30.48	-30.41	8.50E-01	0.0001
GRF5_JAW	COL_JAW	0.82	84.11	0.23	14.85	-30.41	-30.48	-30.41	8.50E-01	0.0001
GRF5_JAW	COL	0.82	84.11	0.23	14.85	-30.41	-30.48	-30.41	8.50E-01	0.0001
DA1_JAW	DA1_JAW	0.92	85.67	0.05	13.36	-34.37	-34.38	-34.37	9.30E-01	0.0001
DA1_JAW	DA1_COL	0.92	85.67	0.05	13.36	-34.37	-34.38	-34.37	9.30E-01	0.0001
DA1_JAW	COL_JAW	0.92	85.67	0.05	13.36	-34.37	-34.38	-34.37	9.30E-01	0.0001
DA1_JAW	COL	0.92	85.67	0.05	13.36	-34.37	-34.38	-34.37	9.30E-01	0.0001
